# Answers to Questions of a Correspondent

**Published:** 1842-03

**Authors:** 


*Answers to Questions of a Correspondent.*—Applicants for admission to membership into the "American Society of Dental Surgeons," must establish by one or more of its members, that they are well informed in the theory and practice of dental surgery, and possessed of an unexceptionable moral character. A written statement to this effect, when the member or members making it are not to be present at the next ensuing meeting, sent or handed to either of the secretaries, or any of the members of the executive committee, will secure to the applicant his election to membership into the Society. But in the absence of this evidence of his worthiness, an examination by a committee appointed for the purpose, and the presentation of samples of his operations are required.

Our opinion in regard to the galvanic action excited by the attaching of clasps and artificial teeth to gold plates by means of gold solder, and also in regard to the substitute proposed by Mr. Brockway is expressed in a note to an article headed "galvanism," by Mr. G. G. Brewster, in the second number of the second volume of the journal, page 191, to which we beg leave to refer our correspondent.

We do not think there are any cases in which the filling of teeth with an *amalgum of mercury and silver,* is justifiable. We have had an opportunity of seeing, perhaps some hundreds of teeth that had been filled with it, and we do not recollect to have met with a single instance where it had been used in which some obvious injurious effect had not resulted from it. We have, it is true, met with a few cases, and it has only been a few, where teeth that had been filled with it but a short time, in which no direct injurious action had been produced upon the subjacent bone, but even in these cases, it had obviously exerted a vitiating influence upon the fluids of the mouth and given rise to an unhealthy action in the gums.—*Balt. Ed.*

